# Prognostic and therapeutic significance of a novel ferroptosis related signature in colorectal cancer patients

**DOI:** 10.1080/21655979.2021.2017627

**Published:** 2022-01-22

**Authors:** Songtao Du, Furong Zeng, Huiyan Sun, Yanlong Liu, Peng Han, Bomiao Zhang, Weinan Xue, Guangtong Deng, Mingzhu Yin, Binbin Cui

**Affiliations:** a Department of Colorectal Surgical Oncology, The Tumor Hospital of Harbin Medical University, Harbin, China; b Department of Dermatology, Hunan Engineering Research Center of Skin Health and Disease, Hunan Key Laboratory of Skin Cancer and Psoriasis, Xiangya Hospital, Central South University, Changsha, Hunan, China; c Department of Oncology, Xiangya Hospital, Central South University, Changsha, Hunan, China; d National Clinical Research Center for Geriatric Disorders, Xiangya Hospital, Central South University, Changsha, Hunan, China

**Keywords:** Ferroptosis-related genes, colorectal cancer, signature, rsl3, fludarabine phosphate

## Abstract

Increasing studies have highlighted the importance of ferroptosis in colorectal cancer (CRC). However, how to use ferroptosis-related genes (FRGs) to predict the prognosis and guide the treatment of CRC remains unknown. To build a prognostic prediction model using the GEO and TCGA databases and explored a therapeutic strategy for CRC patients based on FRGs. A total of 60 FRGs were identified and three of them including ACACA, GSS, and NFS1 were associated with the prognosis of CRC. Using Lasso regression analysis, an FRGs signature was constructed and validated as an independent prognostic predictor. Then we developed a nomogram based on the FRGs signature and clinical prognostic factors to predict the prognosis of CRC patients, which was better than the traditional TNM staging system. Single-sample gene set enrichment analysis (ssGSEA) was further performed for the functional analysis and suggested that JAK-STAT signaling, Ras signaling pathway, MAPK signaling pathway, and PI3K-Akt signaling pathway were significantly enriched in CRC patients with higher FRGs risk score. Interestingly, CRC cells with higher FRGs risk score were more sensitive to RSL3. Knocking down GSS and NFS1 increased the FRGs risk score and the sensitivity of CRC cells to RSL3. For the clinic use, we screened 75 FDA-approved cancer drugs and found that Fludarabine phosphate could decrease the expression of GSS and NFS1 most. Fludarabine phosphate, in combination with RSL3, showed a strong synergistic effect on CRC cells. Together, this study identified a potent prognostic model and provided an alternative individualized treatment for CRC patients.

## 1. Introduction

Colorectal cancer (CRC) is one of the most frequently occurring malignant gastrointestinal tumors, and it remains the second leading cause of cancer-related mortality worldwide, with an estimated 1.4 million new cases and 693, 900 related deaths in 2020 [1,2]. The combination of curative resection and adjuvant chemotherapy has become the standard therapeutic method and achieved a significant improvement in the overall prognosis of CRC [3]. However, a series of epigenetic and metabolic changes confer high migration and invasion capabilities in colorectal cancer cells [4], leading to a 5-year survival rate of approximately 12% for metastatic colorectal cancer [5]. For decades, the American Joint Commission on Cancer/International Union against Cancer tumor-node-metastasis (TNM) staging system has been the gold standard for estimating the prognosis of colorectal cancer. However, many patients at the same stage have varied clinical prognoses, and even patients with stage IIb tumors tend to have a poorer prognosis than those with stage IIIa tumors [6], suggesting that the TNM staging system has shortcomings in the prediction of CRC prognosis. Therefore, an approach combining the TNM staging system and other prognostic stratification parameters is desperately needed to guide decision-making and reveal personalized treatment strategies.

Ferroptosis was recently recognized as a type of regulated cell death marked by the iron-dependent accumulation of lipid hydroperoxides and has emerged as an essential factor in tumor biology [7]. Dysregulation of ferroptosis has been implicated in various disease states such as neurodegeneration, ischemia-reperfusion injury, and cancer [8]. Increasing studies have highlighted the importance of ferroptosis in the development and treatment of CRC [9,10]. For example, Wei et al. reported that activation of p53 with small molecule compounds induced strong inhibitory activity against HCT116 cells via ferroptosis [11]. Lee and his colleagues showed that combinatorial treatment with ferroptosis agents and tumor necrosis factor related apoptosis inducing ligand resulted in synergistic apoptosis and growth regression of CRC [12].

Given the significance of FRGs in CRC patients [13,14], ferroptosis-related genes (FRGs) may hold great promise as therapeutic targets and prognostic predictors in CRC. In this study, we aimed to use FRGs to predict the prognosis and guide the treatment of CRC. We developed and validated an FRGs signature based on three prognostic FRGs (ACACA, GSS, and NFS1) to predict the prognosis of CRC patients. Using the FRGs signature and clinical prognostic factors, we constructed and validated a prognostic nomogram, which was better than the traditional TNM staging system. To clarify the potential mechanism that FRGs signature acts as an independent risk factor for the prognosis of CRC, single-sample gene set enrichment analysis (ssGSEA) was performed and suggested that JAK-STAT signaling, Ras signaling pathway, MAPK signaling pathway, and PI3K-Akt signaling pathway were significantly enriched in CRC patients with higher FRGs risk score. Interestingly, we found that CRC cells with higher FRGs risk score were more sensitive to ferroptosis, which was validated by knockdown GSS and NFS1 expression. Through drug screening, we found that fludarabine phosphate decreased GSS and NFS1 expression most and showed a strong synergistic effect with RSL-3 on CRC cells. Together, this study identified a potent prognostic model and provided an alternative individualized treatment for CRC patients.

## 2. Materials & Methods

### 2.1 Data collation and analysis

The transcriptome data and clinical data were collected from a publicly available dataset (GSE39582) from the NCBI Gene Expression Omnibus (GEO) database (https://www.ncbi.nlm.nih.gov/geo/query/acc.cgi?acc=GSE39582) and The Cancer Genome Atlas (TCGA) database (https://cancergenome.nih.gov/). The mRNA expression profiles of 556 colon cancer patients from the GSE39582 dataset were utilized as the training set, and those of 287 colon cancer patients from the TCGA dataset were selected as the validation dataset. Individuals who meet the following criteria were included: (1) integrated pathological diagnosis of colon cancer, (2) available mRNA expression data, and (3) available survival information. Sixty FRGs assessed were summarized in Zhang’s study [15], and the ferroptosis pathway (map04216) was obtained from the Kyoto Encyclopedia of Genes and Genomes (KEGG) pathway.

### 2.2 Identification and construction of the FRGs signature

Eighteen FRGs were first screened out by univariate Cox analysis (P < 0.05). Three of them (ACACA, GSS, and NFS1) were still associated with the prognosis of CRC patients (P < 0.01). Lasso Cox regression model was applied to obtain the regression coefficients for these crucial candidate genes for the signature. Kaplan-Meier survival analysis was performed to compare and analyze the overall survival of those with high and low signature scores. The predictive FRGs signature was validated externally with the same formula and cut off in the TCGA dataset.

### 2.3 Functional enrichment analysis

The molecular functions of FRGs were investigated by Gene Ontology (GO) analysis and KEGG pathway analysis via the Database for Annotation, Visualization, and Integrated Discovery (DAVID) (https://david.ncifc rf.gov/). The protein-protein interaction (PPI) network of the FRGs was explored with the Search Tool for the Retrieval of Interacting Genes/Proteins (STRING) database (https://stringdb.org). The immunological biological functions of the prognostic genes in high-risk and low-risk patient groups were analyzed by ssGSEA.

### 2.4 Risk model and clinical characteristics analysis

Multivariate cox regression analysis was performed to identify independent risk factors, including sex, age, TNM stage, and FRGs score. All the independent risk factors were enrolled into construct a nomogram including clinical data and the risk score. The area under the curve (AUC), concordance index (C-index), receiver operating characteristic (ROC), and calibration plots were used to confirm the performance of the nomogram.

### 2.5 Cell culture and transfection

The CRC cell lines SW480 and Caco2 were purchased from Xiangya Cell Line Library (Hunan, China), and the human colorectal epithelial cell line NCM460 was obtained from the Myocardial Ischemia Laboratory, affiliated with Harbin Medical University. All the cell lines were cultured in 1640 medium (BI, Israel) supplemented with 10% fetal bovine serum (BI, Israel) and 1% penicillin-streptomycin (BI, Israel). All cells were incubated at 37°C in humidified air with 5% CO_2_. Transfection of siRNA (GenePharma, China) was performed according to the manufacturer’s instructions. A nontargeting siRNA was used as a control. The siRNA sequences are shown in Supplement Table 1.

### 2.6 Drugs and inhibitors

An inducer of ferroptosis [a specific GPX4 inhibitor 1S,3 R-RSL3 (T3646)], inhibitors of ferroptosis [ferrostatin (Fer-1; T6500), deferoxamine mesylate (DFO; T1637)], an inhibitor of apoptosis [Z-VAD(OMe)-FMK (Z-VAD; T6013)] and an inhibitor of necrosis [necrostatin-1 (Nec-1; T1847)] were purchased from Target Mol (China, Shanghai). ML210 (S0788) and 75 FDA-approved drugs were purchased from Selleck. Drug list is shown in Supplement Table 2.

### 2.7 Cell viability assays

Cell viability was assessed using a Cell Counting Kit-8 (CCK-8) assay (Bimake, USA) according to the manufacturer’s instructions. Cells were seeded into 96-well plates at 6,000 cells/well and cultured for 24 h. The 96-well plate contained with small molecule drugs was incubated for 18, 24, 36, and 48 h. Finally, the absorbance of each well was measured using a spectrophotometer (Beckman, USA) at an emission wavelength of 450 nm. The combination index (CI) scores for Loewe additivity were acquired using the Ting Chao Chou CI-isobologram equation applied in CompuSyn (http://www.combosyn.com/) [16].

### 2.8 Total RNA extraction and quantitative real-time PCR

Total cellular RNA was isolated using MagZolReagent (R4801-01, Magen) and Genomic DNA contamination was eliminated by treatment with DNase I (R223-01 Vazyme). First-strand cDNA synthesis was synthesized with a HiScript II Q RT SuperMix for qPCR Kit (R223-01 Vazyme) according to the manufacturer’s protocol. Quantitative real-time PCR was carried out using Power 2x SYBR Green qPCR Master Mix (B21702, Bimake). The reaction was run and analyzed using the QuantStudio 3 Real-Time PCR System (Applied Biosystems). The relative quantitation expression levels of marker genes were normalized to GAPDH. Primers used are listed in Supplemental Table 1.

### 2.9 Lipid ROS assay

Cells in 6-wells plate were incubated with PBS containing 5 μM C-11 BODIPY probes (Invitrogen, D3861) for 30 min. Then cells were harvested and washed twice with PBS. We used the Cytek Athena flow cytometry system to analyze the lipid ROS levels in FITC channel, and the data were analyzed by FlowJo 10. Three hundred thousand or more cells were analyzed for each sample.

### 2.10 Statistical analysis

The ‘clusterProfiler’ R package was utilized to conduct GO and KEGG analyses. Univariate and multivariate Cox regressions were implemented to identify independent predictors of OS by using the ‘survival’ and ‘survminer’ package in R. ROC analyses and the subsequent calculation of AUC were performed using the ‘survivalROC’ package. The immune infiltrating score of 16 immune cells and the activity of 13 immune-related pathways in the tumor microenvironment (TME) were calculated by calculating ssGSEA with the application of the ‘GSVA’ ‘GSEAbase’ and ‘ggpubr’ package in R. The values for each experiment are representative of at least three independent experiments. P < 0.05 was considered statistically significant.

## 3. Results

### 3.1 Screening of FRGs associated with the prognosis of CRC patients

**Figure 1.**
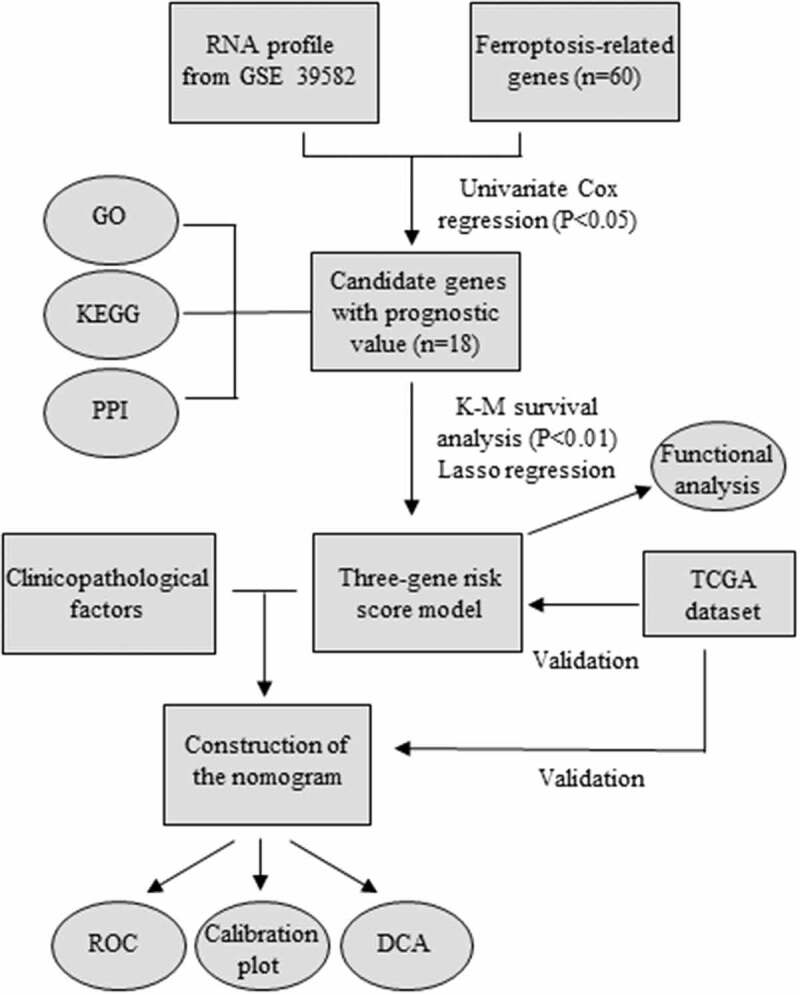
A flowchart of the study.

**Figure 2.**
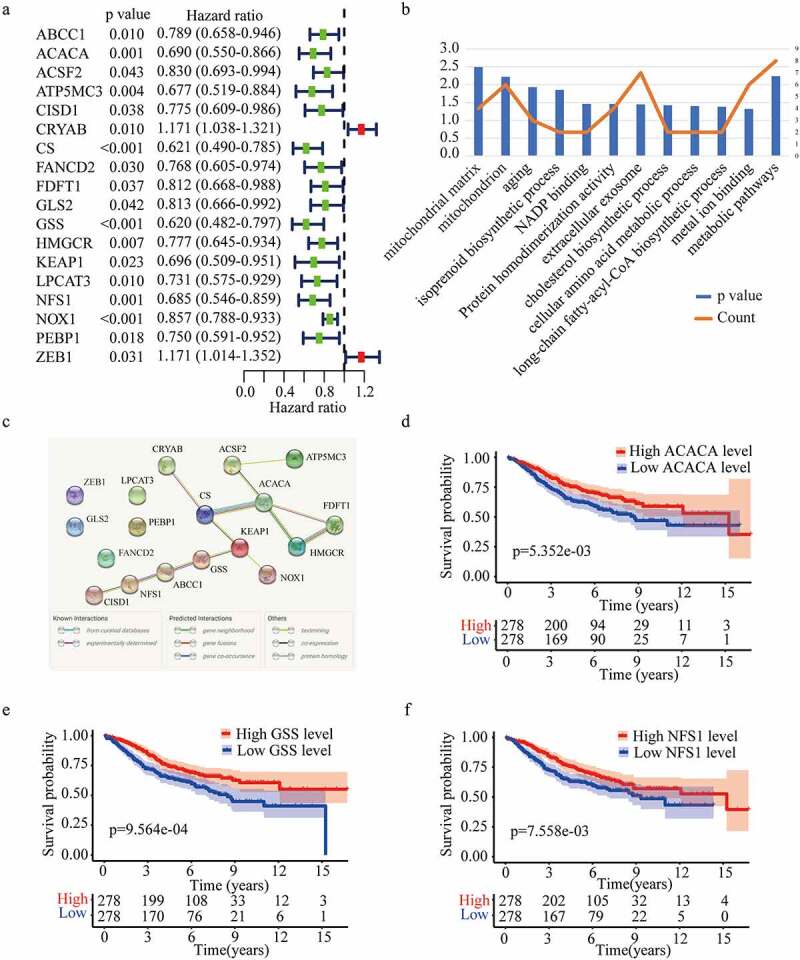
**Identification of optimal prognostic ferroptosis-related genes in GSE 39582**. (a) Univariate Cox regression analysis of gene expression and overall survival. (b) Gene ontology (GO) terms and Kyoto Encyclopedia of Gene and Genomes (KEGG) pathways of the 18 candidate genes. (c) Interaction among the candidate genes analyzed through protein-protein interactions (PPI) network. (d-f) Kaplan-Meier survival curves for the overall survival in the high and low expression group of ACACA (d), GSS (e), and NFS1 (f) in GSE 39582.

Considering the importance of FRGs in CRC patients, targeting ferroptosis-related genes may provide therapeutic targets and prognostic predictors. As the flow chart showed (Figure 1), we aimed to use FRGs to predict the prognosis and guide the treatment of CRC. To explore the function of FRGs in CRC patients, in the GSE39582 training cohort, we identified 18 ferroptosis-related genes related to the prognosis of CRC patients based on univariate Cox regression analysis (P < 0.05) (Figure 2a). GO and KEGG pathway analyses showed that these FRGs were mainly enriched in cholesterol biosynthetic process, cellular amino acid metabolic process, and metabolic pathways (Figure 2b). The protein-protein interaction network among the 18 genes was established using the STRING online platform (Figure 2c). To further evaluate the prognostic significance of these genes, K-M survival curves were drawn and only three genes were significantly associated with the prognosis of CRC patients including ACACA, NFS1, and GSS (P < 0.01) (Figure 2 **(d-f**)).

### 3.2 Construction and validation of FRGs signature

**Figure 3.**
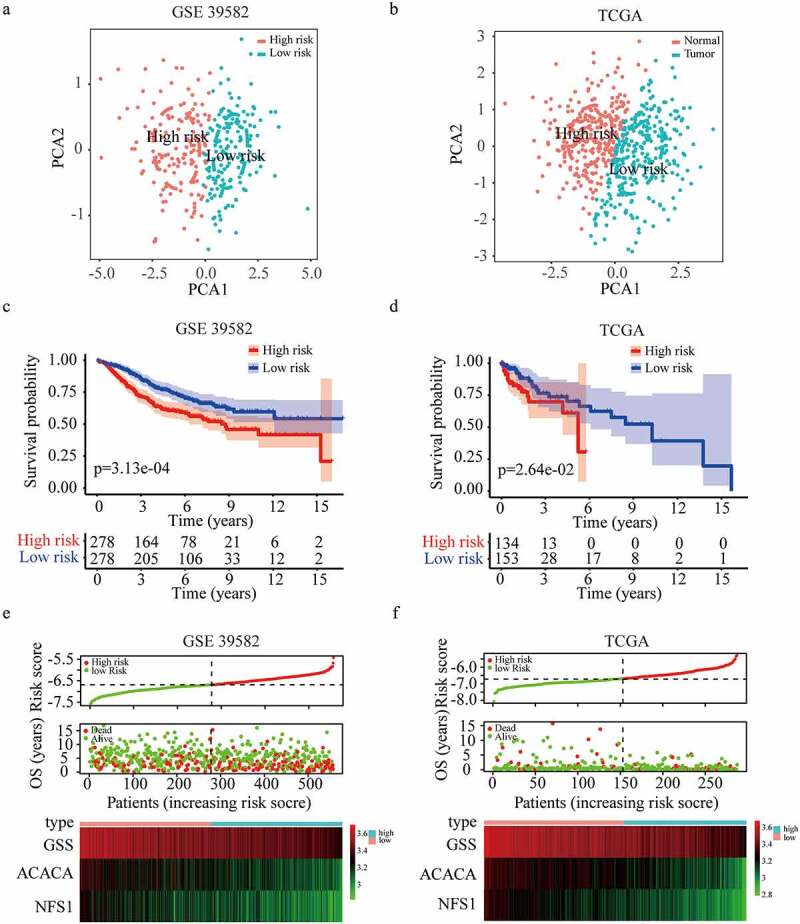
**Construction and validation of the three-gene risk score model.** (a-b) Principal component analysis plot of GSE 39582 (a) and TCGA dataset (b). (c-d) Kaplan-Meier survival curves for the overall survival in high and low-risk group in GSE 39582 (c) and TCGA dataset (d). (e-f) The distribution of risk score, overall survival status, and gene expression profile in GSE 39582 (e) and TCGA dataset (f).

Using these three genes, a prognostic FRGs signature was constructed through Lasso regression analysis. The risk score of each patient was assessed with the following formula = (−0.295 × expression of ACACA) + (−0.158 × expression of NFS1) + (−0.289 × expression GSS). Based on the same formula and median cutoff value (−6.69), patients in training (GSE39582) and validation (TCGA) cohorts were evenly distributed into high or low groups. Principal component analysis showed that high-risk patients were clearly differentiated from those at low risk in both cohorts (Figure 3(a- b)). Notably, patients in the high-risk group had a poorer prognosis than those in the low-risk group based on the K-M survival analysis in GSE39582 cohort (five-year overall survival rate, 60.7% vs. 75.3%) and TCGA cohort (five-year overall survival rate, 30.5% vs. 66.3%) (Figure 3(c-d)). The distributions of the risk score, survival status, and a heatmap of the gene expression profile are presented in Figure 3e **and** 3f, suggesting that patients in the high-risk group had lower expressions of GSS, ACACA, and NFS1, and more death cases.

### 3.3 Identification of FRGs signature as an independent prognostic predictor

**Figure 4.**
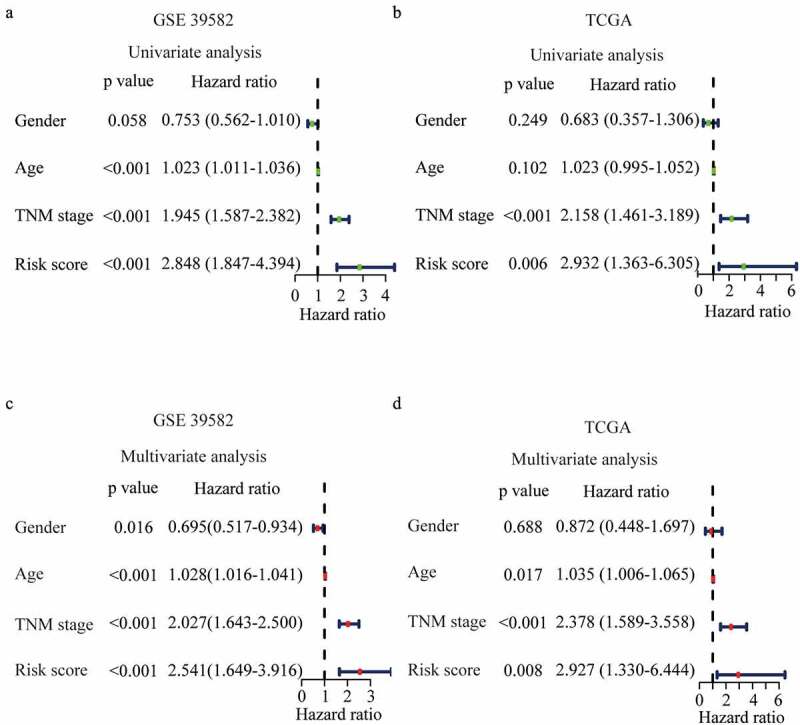
**Prognostic performance of the risk score model.** (a-b) Univariate analysis Cox regression analysis of the risk score model and clinico-pathological factors in GSE 39582 (a) and TCGA dataset (b). (c-d) Multivariate analysis Cox regression analysis of the risk score model and clinicopathological factors in GSE 39582 (c) and TCGA dataset (d).

To verify the predictive effect of the FRGs signature on the prognosis of CRC, we performed univariate Cox regression analyses and results showed that FRGs signature was associated with the poor prognosis in training (HR = 2.848, 95% CI = 1.847–4.394, P < 0.001) and validation (HR = 2.932, 95% CI = 1.363–6.305, P = 0.006) cohort (Figure 4(a-b)). To explore the independent prognostic predictor of CRC, FRGs signature and clinic parameters were entered into multivariate Cox regression analyses. In the training cohort, independent prognostic predictors for CRC were found to be gender (HR = 2.541, 95%CI 1.649–3.916), FRGs signature (HR = 2.541, 95%CI 1.649–3.916), age (HR = 0.695, 95%CI 0.517–0.934) and TNM stage (HR = 2.027, 95%CI 1.643–2.500) (Figure 4c). In the validation cohort, the FRGs signature was further validated as an independent prognostic predictor (HR = 2.541, 95%CI 1.649–3.916) (Figure 4d).

### 3.4 Establishment and validation of an FRGs nomogram

**Figure 5.**
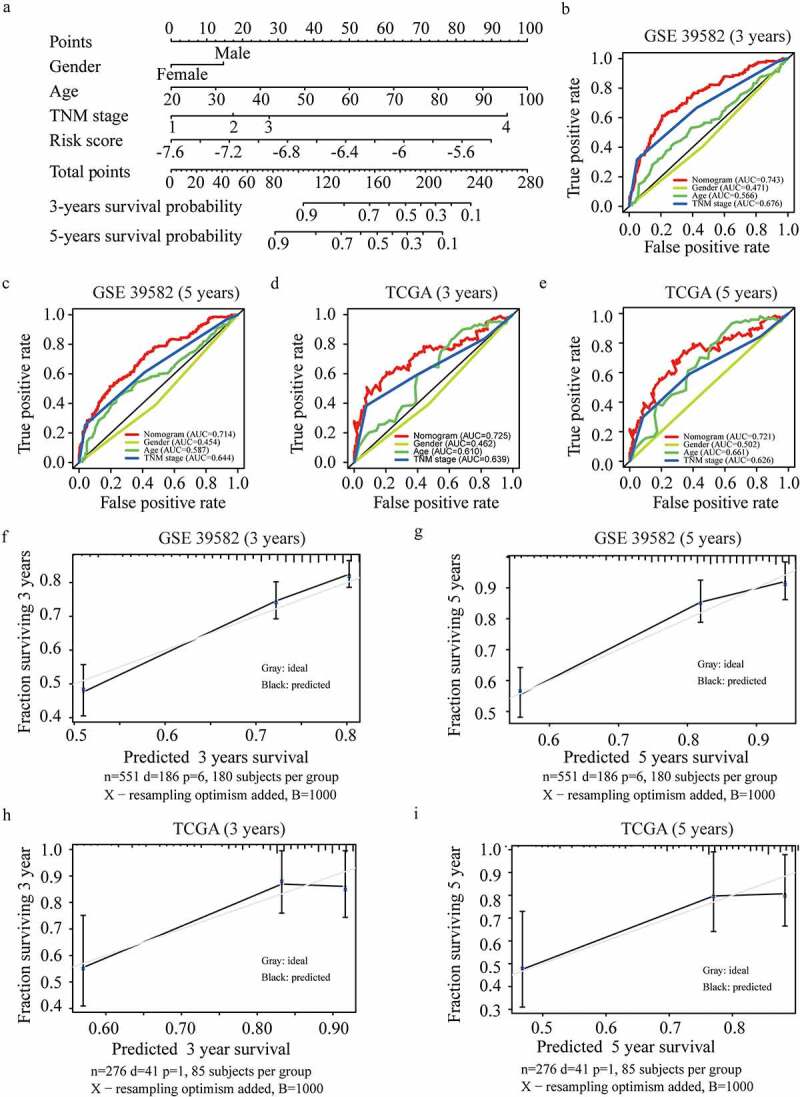
**Development and validation of the nomogram.** (a) Development of the nomogram. (b-c) ROC curves of the nomogram, gender, age, and TNM stage at 3 (b) and 5 years (c) in GSE 39582. (d-e) ROC curves of the nomogram, gender, age, and TNM stage at 3 (d) and 5 years (e) in the TCGA validation dataset. (f-i) Calibration plots for 3 (f) and 5 years (g) overall survival prediction in GSE 39582, and for 3 (h) and 5 years (i) overall survival prediction in TCGA validation dataset.

Clinically, patients with the same stage have varied clinical prognoses, suggesting a need for a more accurate predictive model [17]. To further optimize the predictive model, all predictors including the risk score, age, gender, and TNM stage, were extracted to establish a nomogram to predict the survival probability at 3 and 5 years (Figure 5a). The practicability of the nomogram was proven by assessing the area under the ROC curve, and the C index of the nomogram was 0.743 for 3-year OS and 0.714 for 5-year OS in the training cohort (Figure 5(b-c)). Moreover, the ROC curve also showed a favorable predictive ability for the 3-years OS rates in the validation dataset (AUC = 0.725, 0.721 for 3-year and 5-year OS respectively) (Figure 5(d-e)). The calibration curve results showed an excellent match with nomogram prediction and the actual survival rate in the GEO training cohort and the TCGA validation cohort (Figure 5(f-i)). The findings of the decision curve analysis for both cohorts indicated that our nomogram might perform better than other models in predicting the survival of CRC patients (**Figure S1 (a-b)**).

### 3.5 Functional analyses of the FRGs signature in CRC

**Figure 6.**
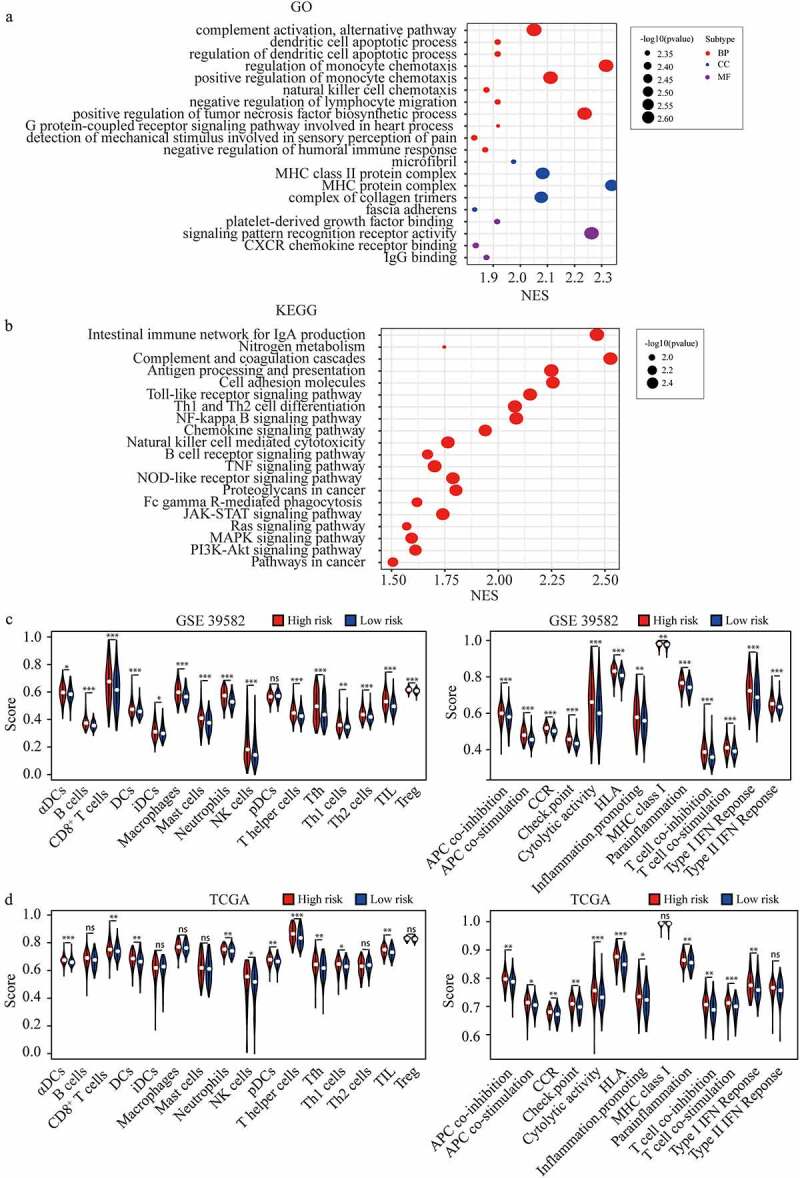
**Pathway analysis and ssGSEA scores comparison between the high and low-risk groups.** (a-b) GO terms (a) and KEGG pathway analysis in GSEA enrichment. (c-d) The scores of 16 immune cells and 13 immune-related functions in high and low-risk group in GSE 39582 (c) and TCGA dataset (d).

To clarify the potential mechanism that FRGs signature affects the prognosis of CRC, we analyzed the GO gene sets and KEGG gene sets between high-risk and low-risk groups using ssGSEA [15] (Figure 6(a-b)). We found that JAK-STAT signaling, Ras signaling pathway, MAPK signaling pathway, and PI3K-Akt signaling pathway were significantly enriched in the high-risk group (Figure 6b). These pathways were usually reported to be associated with tumor progression, and could partly explain the reason that higher FRGs signature predicted the poor prognosis of CRC. In addition, we found that many immune-related gene sets were significantly enriched in the high-risk group (Figure 6(a-b)). Furthermore, we quantified the enrichment scores of diverse immune cell subpopulations, related functions, or pathways with ssGSEA, most of which were enriched in the high-risk group in both cohorts (Figure 6(c-d)). These finding suggested that FRGs signature was highly associated with immunity and further researches were needed to clarify the association.

### 3.6 Higher FRGs risk score predicts more sensitivity to ferroptosis-inducer

**Figure 7.**
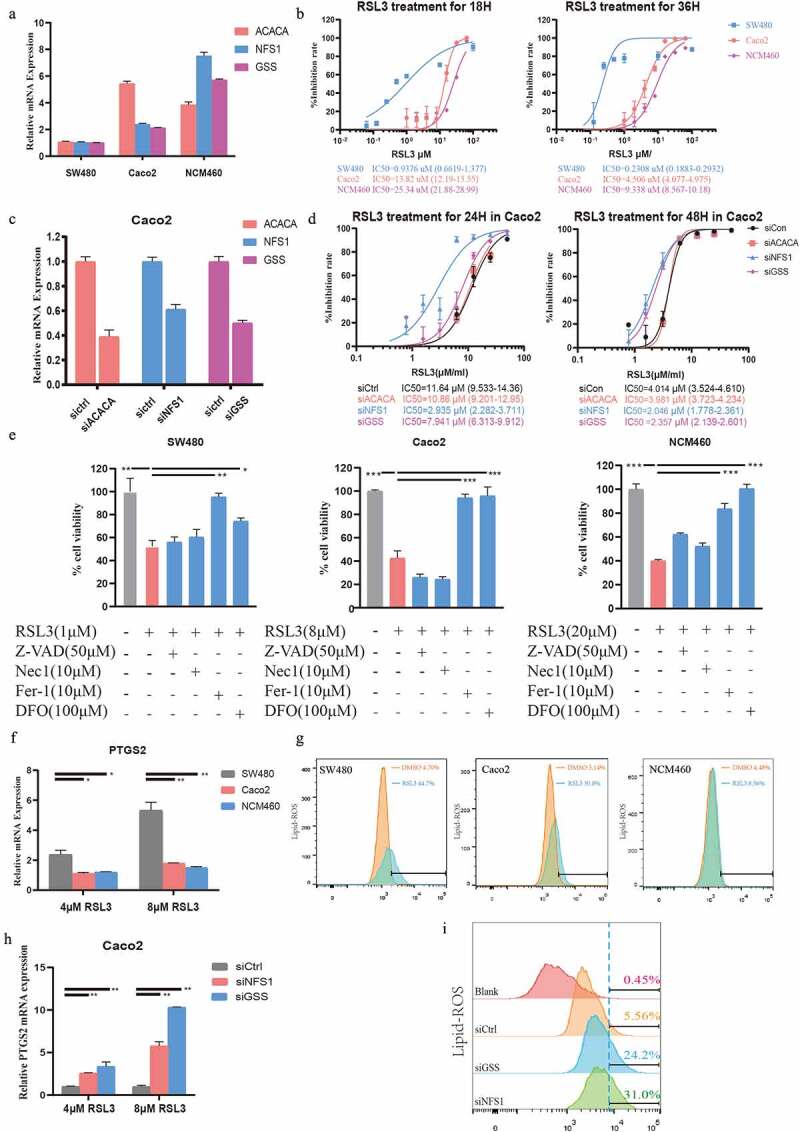
**Higher FRGs signature predicts more sensitivity to ferroptosis-inducer.** (a) The expression of ACACA, NFS1 and GSS in SW480, Caco2 and NCM460 cells (GAPDH as acommon reference). (b) Cell viability was assayed by CCK-8 kit after treatment with RSL3 (0, 1.5625, 3.125, 6.25, 12.5, 25, and 50 μM) for 18 or 36 hrs. (c) Knockdown efficiency of ACACA, NFS1, and GSS in Caco2 cells transfected with siRNAs. (d) Cell viability of Caco2 cells transfected with siCtrl, siACACA, siGSS or siNFS1 for 48 hrs and then treated with RSL3 (0, 1.5625, 3.125, 6.25, 12.5, 25, and 50 μM) for 18 or 36 hrs. (e) Cell viability of the indicated CRC cells after treatment with RSL3 in the absence or presence of Z-VAD-FMK(Z-VAD), Necrostatin-1(Nec1), ferro-statin-1(Fer-1), and Deferoxamine Mesylate (DFO) for 24 hrs (n = 5, *p < 0.05). (f, h) Quantitative polymerase chain reaction (qPCR) analysis of the PTGS2 expression in the indicated cells treated with RSL3 (4 or 8 μM) for 24 hrs. (g, i) Identification by flow cytometry of C11-BODIPY fluorescence in SW480, Caco2 or NCM460 cells (g), and in Caco2 cell transfected with siCtrl, siGSS or siNFS1 (i) after treatment with RSL3 (2 or 4 μM) for 24 hrs.

Considering that the FRGs signature was an independent prognostic risk factor, we wondered whether the FRGs signature was associated with ferroptosis-related therapy. We firstly checked the mRNA levels of ACACA, NFS1, and GSS in different CRC cell lines and normal colon cell line by RT-PCR and calculated their FRGs signature based on the formula mentioned above (Figure 7a). Interestingly, the SW480 cell line with the highest FRGs signature was most sensitive to RSL3, a ferroptosis inducer, while NCM460, a normal intestinal epithelial cell line, with the lowest FRGs signature was insensitive to RSL3 (Figure 7b). To further clarify the association of FRGs signature and ferroptosis inducers, we silenced ACACA, NFS1, and GSS in the Caco2 cell line (Figure 7c), and found that cells with knockdown of NFS1 or GSS were more sensitive to RSL3, while knockdown ACACA did not change the sensitivity of Caco2 cell line to RSL3 (Figure 7d). In order to check whether ferroptosis is induced by RSL3 in CRC cell lines, different ferroptosis inhibitors were used under RSL3 treatment. As expected, ferroptosis inhibitors such as Fer-1 or DFO, but not apoptosis or necrosis inhibitors, could block the death induced by RSL3 treatment (Figure 7e). PTGS2, a marker for ferroptosis, is highly increased under the RSL3 treatment (Figure 7f) [18]. What’s more, lipid ROS was also increased after RSL3 treatment, especially in SW480 cells (with the highest FRGs signature) (Figure 7g). Expression of PTGS2 and lipid ROS were further increased in GSS or NFS1 knockdown Caco2 cells after RSL3 treatment (Figure 7(h-i)). These findings suggested that cells with higher FRGs signature were more sensitive to ferroptosis-inducers.

       

### 3.7 Fludarabine phosphate has a synergistic effect in combination with ferroptosis-inducer

**Figure 8.**
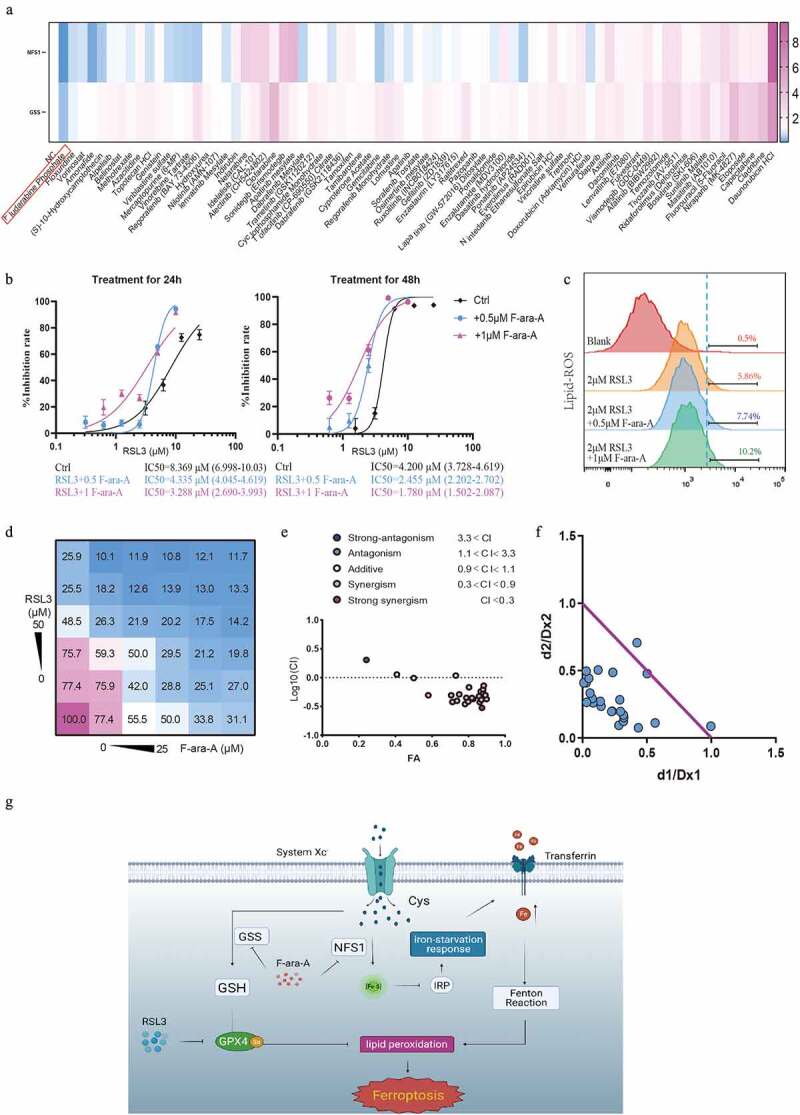
**Fludarabine phosphate has a synergistic effect in combination with RSL3.** (a)Heat map of mRNA expression levels of NFS1 and GSS in Caco2 cell lines following treatment of 78 FDA-approved drugs for 48hours (The working concentrations were determined based on the Selleck website). (b) Cell viability of Caco2 cells after treatment of 0.5μM or 1μM Fludarabine Phosphate combined with RSL3 (0, 0.625, 1.25, 2.5, 5 and 10μM) for 24 or 48 hr. (c) Identification by flow cytometry of C11-BODIPY fluorescence in Caco2 treated with 2μM RSL3 combined with 0.5μM or 1μM Fludarabine Phosphate for 24 hr. (d) Heatmap showing the cell viability of Caco2 cell after RSL3 and Fludarabine Phosphate combination treatment. (e)Synergy of RSL3 and the Fludarabine Phosphate assessed by TING-CHAO CHOU combination index (CI). The x-axis represents inhibi-tion effect, and the yaxis represents log10(CI, Combination Index). ACI of less than 1 means that the two drugs have asynergistic effect. (f) The horizontal and vertical co-ordinates represent the fractional inhibition effect of the two drugs (Dx1 (RSL3), and Dx2 (Fludarabine Phosphate)) standardized concentrations alone. The purple line dis-played is the line of additivity. (g) The working model depicting the synerg**i**stic effect of RSL3 and the Fludarabine Phosphate on CRC cells.

We have confirmed that down-regulating NFS1 and GSS could increase the sensitivity of CRC cells to ferroptosis-inducers (Figure 7d). To identify the drugs which could have a synergetic effect with ferroptosis-inducers, we screened 75 FDA-approved cancer drugs and found that fludarabine phosphate (F-ara-A) inhibited NFS1 and GSS expression most (Figure 8a). Additionally, F-ara-A sensitized colon cells to RSL3 in a dose-dependent manner (Figure 8b). Similarly, the same trend was presented in the lipid-peroxidation assay. The addition of F-ara-A increased the proportion of ferroptosis cells, which suggested the F-ara-A enhanced the sensitivity of colorectal cancer to ferroptosis (Figure 8c). Moreover, the isobologram and combination index (CI) was calculated based on combination theory with CI<0.9 representing synergy [16]. We found that there was a strong synergistic effect between F-ara-C and RSL3, with an average CI of 0.67 (Figure 8(d-f)). In summary, F-ara-A could sensitize CRC cells to RSL-induced ferroptosis by downregulating NFS1 and GSS, which provide a potential therapeutic avenue for CRC (Figure 8g).

## 4. Discussion

Ferroptosis is a new nonapoptotic form of cell death characterized by lipid reactive oxygen species accumulation [7]. The most relevant mechanisms regulating the ferroptosis were amino acid and lipid metabolism [8]. Amino acids and lipid levels impact tumor cell pathologic behavior (proliferation or death), resulting in gene-dependent changes in the stimulation response [19]. Moreover, the drug resistance and exist of colorectal cancer stem cells pose a major challenge in the achievement of lower mortality and effective therapeutic strategy [20,21]. Notably, many studies had confirmed that ferroptosis presents a new therapeutic avenue for drug-resistance cells [22–24], which highlighted the role of ferroptosis in the treatment of CRC patients.

In this study, we identified three genes related to CRC patient prognosis from 60 ferroptosis-related genes based on the GEO database. ACACA, a protein-coding gene, can encode acetyl-CoA carboxylase 1 that catalyzes the carboxylation of acetyl-CoA to malonyl-CoA. Acetyl-CoA carboxylase 1 is an essential rate-limiting enzyme of fatty acid metabolism and the biosynthesis of polyunsaturated fatty acids [25,26] and polyunsaturated ether phospholipids, the main substrates for lipid peroxidation and the induction of ferroptosis [27]. Undoubtedly, ACACA is of great significance in ferroptosis. GSS, encoding glutathione synthetase, regulates the synthesis of glutathione, which is an essential component of the reactive oxygen species scavenging system [28]. NFS1 delivers sulfur to scaffold protein iron-sulfur clusters and plays roles in DNA maintenance, protein translation, and energy conversion. Richard Possemato et al. reported that suppression of NFS1 could trigger iron starvation and promoted ferroptosis when cells encountered ROS [29]. These findings suggested that these three genes are important for the regulation of ferroptosis.

Based on these three genes, we built a signature to predict the prognosis of CRC patients. The FRGs signature shows certain predictive feasibility in both training and validation cohorts, and the risk score of FRGs signature is an independent prognostic indicator of overall survival. ssGSEA was performed to explore the potential mechanism of how FRGs signature affects the prognosis of CRC, and we found that some pathways such as JAK-STAT signaling and Ras signaling pathway, were significantly enriched in CRC patients with higher FRGs risk score. Moreover, the degree of immune cell infiltration and their expression levels in the two groups were different. We found that the high-risk group was with higher immune scores and relatively lower tumor purity. The detailed mechanism needs further investigations.

Many previous studies had highlighted the importance of ferroptosis in predicting tumor prognosis. For example, Qian and his colleagues reported a ferroptosis-related predictive model in papillary thyroid carcinoma patients [30]. Qi et al. constructed a novel prognostic signature based on FRGs in cervical cancer [31]. However, these studies didn’t investigate their implications for cancer treatment. Here, we further explored the role of FRGs signature on CRC therapy. We found that CRC cell lines with low expression of the genes in FRGs signature, GSS or NFS1, were more sensitive to RSL-induced ferroptosis. The reasons might be that downregulation of GSS could affect the synthesis of glutathione [32], while NFS1 might affect the cell sensitivity to ferroptosis through regulating the synthesis of Fe-S clusters in the iron metabolic pathway [29,33]. We next screened 75 FDA-approved drugs and identified fludarabine phosphate as a potential clinically applicable inhibitor of GSS and NFS1. Combination of fludarabine phosphate and RSL3 showed a synergistic effect on CRC cell viability. Altogether, our prediction model based on FRGs can not only improve individual prognosis monitoring but also provide a new ferroptosis-related treatment strategy for CRC patients.

Inevitably, there are still some inherent limitations in our research. First, further mechanism studies were needed to uncover the exact role of each gene. Second, some information such as treatment was not involved in our study. Third, an external validation based on prospective and large-scale clinical trials was needed to evaluate the prediction ability of the models.

## 5. Conclusions

In summary, we constructed and validated a prognostic and therapeutic prediction model based on FRGs. Our findings will assist with decision making for clinicians and improve the treatment for CRC patients.

## Supplementary Material

Supplemental Material

## Research highlights

- FRGs signature is an independent prognostic predictor in CRC patients.
- The FRGs-based nomogram functions better than TNM staging system on the prognosis.
- Fludarabine phosphate combined with RSL3 has a synergistic effect on CRC cells.
