# Supplementary material for: Prognostic and therapeutic significance of a novel ferroptosis related signature in colorectal cancer patients
Source: Bioengineered. 2022 Jan 22;13(2):2498–512. doi: 10.1080/21655979.2021.2017627 (PMC8973628; doi:10.1080/21655979.2021.2017627)
Supplement: Supplemental Material [file KBIE_A_2017627_SM0314.zip › supplementary/Supplemental Table 1clean.docx]

Supplemental Table 1. Clinical information of all patients.

| Patient | Gender | Age | Tumor size(≥5cm) | HBV infection | Cirrhosis |
| --- | --- | --- | --- | --- | --- |
| 1 | male | 51 | no | yes | no |
| 2 | female | 45 | no | yes | yes |
| 3 | female | 56 | no | yes | yes |
| 4 | male | 60 | no | no | yes |
| 5 | male | 58 | no | yes | yes |
| 6 | male | 47 | no | yes | yes |
| 7 | male | 55 | yes | yes | yes |
| 8 | female | 54 | no | no | no |
| 9 | male | 59 | no | yes | yes |
| 10 | male | 62 | yes | yes | yes |
| 11 | female | 57 | no | no | yes |
| 12 | female | 54 | no | no | no |
